# Therapeutic targeting of obesity-induced neuroinflammation and neurodegeneration

**DOI:** 10.3389/fendo.2024.1456948

**Published:** 2025-01-17

**Authors:** Jialiu Zeng, Lenny Yi Tong Cheong, Chih Hung Lo

**Affiliations:** ^1^ Department of Biomedical and Chemical Engineering, Syracuse University, Syracuse, NY, United States; ^2^ Interdisciplinary Neuroscience Program, Syracuse University, Syracuse, NY, United States; ^3^ Lee Kong Chian School of Medicine, Nanyang Technological University, Singapore, Singapore; ^4^ Department of Biology, Syracuse University, Syracuse, NY, United States

**Keywords:** obesity, metabolic dysfunction, neuroinflammation, neurodegeneration, body-brain interactions, therapeutic targeting

## Abstract

Obesity is a major modifiable risk factor leading to neuroinflammation and neurodegeneration. Excessive fat storage in obesity promotes the progressive infiltration of immune cells into adipose tissue, resulting in the release of pro-inflammatory factors such as cytokines and adipokines. These inflammatory mediators circulate through the bloodstream, propagating inflammation both in the periphery and in the central nervous system. Gut dysbiosis, which results in a leaky intestinal barrier, exacerbates inflammation and plays a significant role in linking obesity to the pathogenesis of neuroinflammation and neurodegeneration through the gut-brain/gut-brain-liver axis. Inflammatory states within the brain can lead to insulin resistance, mitochondrial dysfunction, autolysosomal dysfunction, and increased oxidative stress. These disruptions impair normal neuronal function and subsequently lead to cognitive decline and motor deficits, similar to the pathologies observed in major neurodegenerative diseases, including Alzheimer’s disease, multiple sclerosis, and Parkinson’s disease. Understanding the underlying disease mechanisms is crucial for developing therapeutic strategies to address defects in these inflammatory and metabolic pathways. In this review, we summarize and provide insights into different therapeutic strategies, including methods to alter gut dysbiosis, lifestyle changes, dietary supplementation, as well as pharmacological agents derived from natural sources, that target obesity-induced neuroinflammation and neurodegeneration.

## 1 Introduction

**Figure 1:**
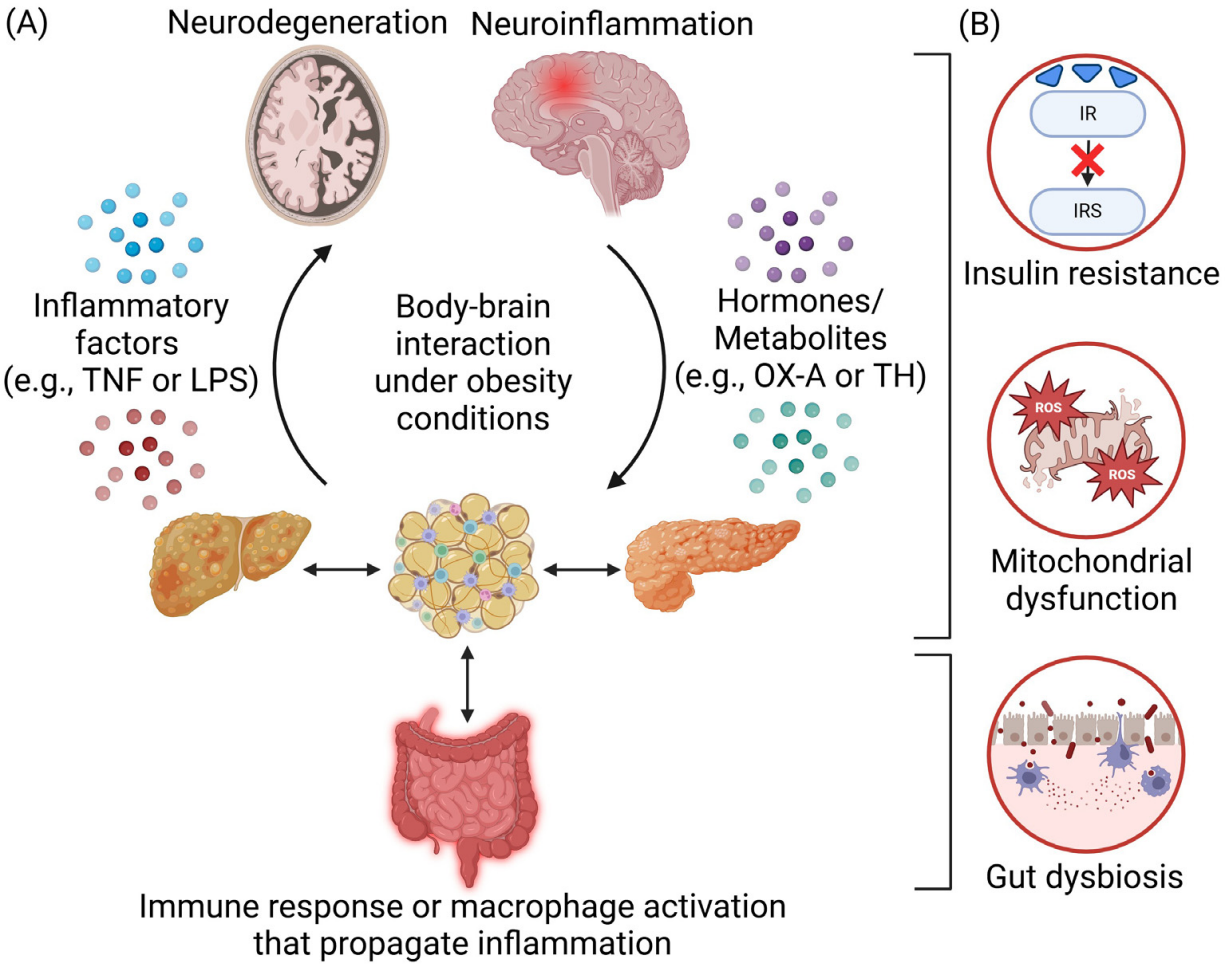
Body-brain interaction under obesity conditions. **(A)** Obesity-induced inflammation arises from the adipose tissues and is propagated across different peripheral organs in the body through immune response or macrophage activation. The communication and interaction between the body and the brain are mediated by inflammatory factors such as tumor necrosis factor (TNF) or lipopolysaccharides (LPS) and hormones or metabolites such as orexin-A (OX-A) and thyroid hormone (TH). Overnutrition will lead to neuroinflammation and neurodegeneration in the brain, which could also, in turn, regulate appetite and satiety as well as the extent of obesity. **(B)** Several key disease mechanisms of obesity-induced neurological disorders include insulin resistance, mitochondrial dysfunction, and gut dysbiosis. Created in https://BioRender.com.

Obesity is a metabolic syndrome characterized by lipid accumulation and is commonly associated with low-grade inflammation due to increased infiltration and activation of innate and adaptive immune cells such as macrophages, dendritic cells, mast cells, neutrophils, B cells, and T cells, within peripheral tissues such as adipose tissues (1, 2) and can contribute to neuroinflammation (3, 4). Under obesity conditions, excess free fatty acids (FFAs) decrease the lipid storage capability of adipose tissue, resulting in hypertrophic adipocytes and overproduction of adipokines and cytokines such as tumor necrosis factor (TNF), interferon-γ, interleukin (IL)-1β, IL-6 and IL-18 triggering systemic inflammation (5, 6). Circulating inflammatory factors can further propagate peripheral inflammation and activate macrophages in other organs such as the liver and pancreas, as well as trigger brain inflammation (5, 7) (**Figure 1A**). Obesity condition can further increase BBB permeability, making the brain more vulnerable to inflammation (8). Neuroinflammation is characterized by the activation of microglia and astrocytes (9, 10), along with the release of proinflammatory and neurotoxic mediators within the brain. Initially a protective mechanism, excess neuroinflammation can lead to neuronal dysfunctions (1, 4) and neurodegenerative diseases such as Alzheimer’s (AD), multiple sclerosis, and Parkinson Disease (PD) (11, 12), which currently do not have effective treatments.

**Figure 2:**
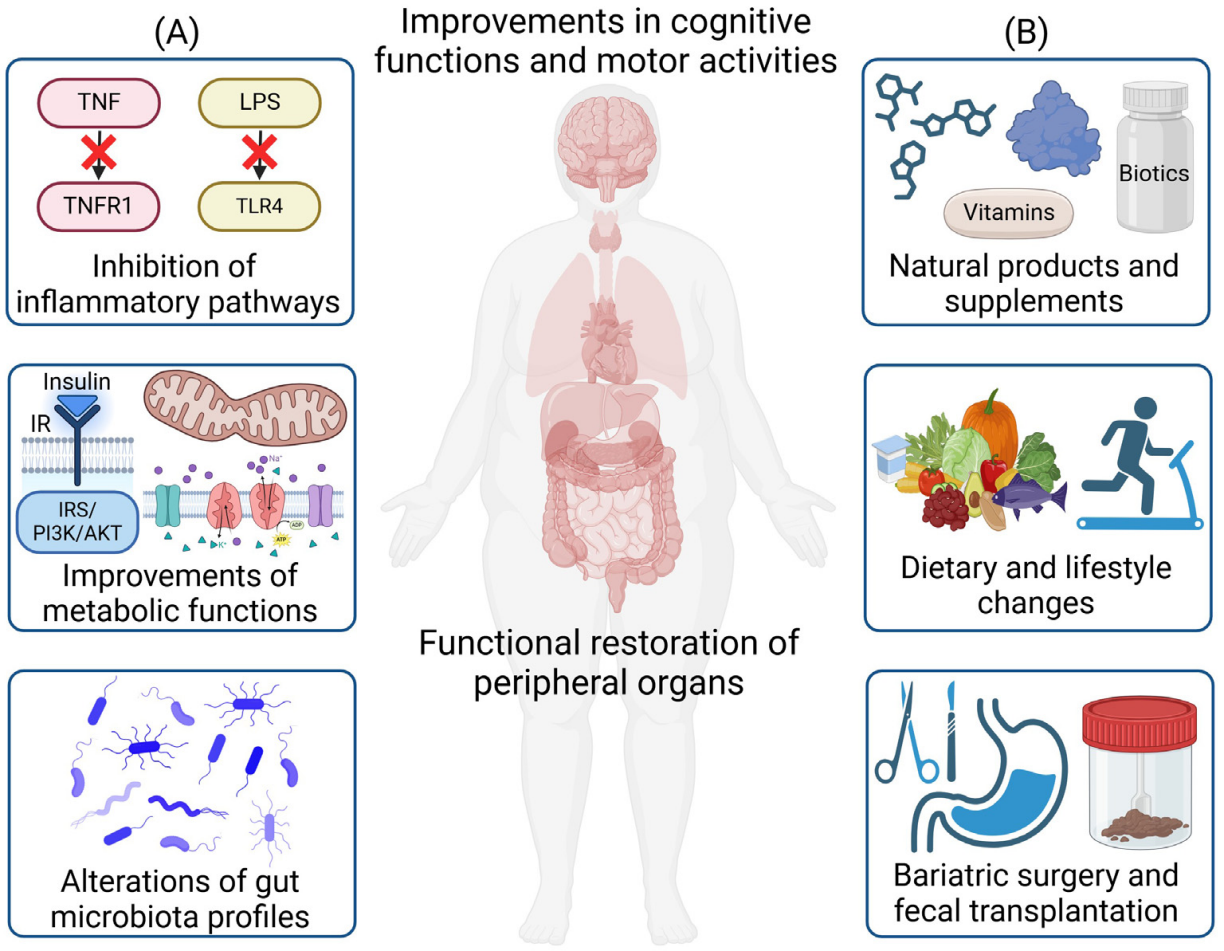
Therapeutic strategies for the treatment of obesity-induced neuroinflammation and neurodegeneration. **(A)** Major disease mechanisms for therapeutic targeting include the inhibition of inflammatory pathways, improvements in metabolic functions, and alterations of gut microbiota profiles. **(B)** Therapeutic strategies include the administration of natural products and supplements, dietary and lifestyle changes, as well as bariatric surgery and fecal microbiota transplantation. These treatments will lead to functional restoration of peripheral organs and the brain, along with improvements in cognitive functions and motor activities. Created in https://BioRender.com.

Understanding the underlying mechanisms governing the pathogenesis of obesity-induced neuroinflammation and neurodegeneration holds the key to discovering novel therapeutics and providing more effective treatments. While body-brain interaction including the gut-brain/gut-liver-brain axis involves the orchestration of multiple organs and cell types (13, 14), a central disease mechanism lies in the crosstalk of inflammation (e.g., cytokines/adipokines production/circulation and gut dysbiosis) and metabolic dysregulations (e.g., insulin resistance, mitochondrial dysfunction, reactive oxygen species (ROS) production, and autolysosomal impairments) (**Figure 1B**). Metabolic dysfunctions are predominant in obesity and neurodegenerative diseases. Obesity can impair insulin signaling that is crucial for hepatic, pancreatic, and neuronal function (15). Inflammatory factors trigger pathways that inhibit insulin receptor (IR) and insulin receptor substrate (IRS) phosphorylation, leading to insulin resistance in the body and brain (16, 17). Under obesity conditions, there are higher levels of mitochondrial fragmentation (18, 19), overproduction of ROS (20), and higher oxidative stress that can further impair mitochondria (21). The autolysosomal pathway is crucial for clearing cellular debris and damaged organelles. Impaired function can lead to excess lipid accumulation, damaged mitochondria, increased ROS production, and inflammation, contributing to obesity in both the body and brain (22, 23). Additionally, obesity-induced gut dysbiosis can increase the intestinal permeability, leading to a “leaky gut” and increased secretion of lipopolysaccharides (LPS), which can further trigger inflammation (24). These factors collectively propagate inflammation throughout the body including the brain (14) (**Figure 2A**).

In this review, our goal is to summarize and provide insights into therapeutic strategies that can mediate obesity-induced neuroinflammation and neurodegeneration through the body-brain axis. In particular, we will focus on a variety of therapies including (1) small molecules and biologics that modulate inflammatory and metabolic pathways, (2) approaches to alter gut microbiota to remedy gut dysbiosis, (3) effect of exercise and diet interventions, (4) vitamins and supplements, and (5) natural products and derivatives (**Figure 2B**). We will further provide future perspectives on emerging therapies as well as the need to further dissect body-brain interaction through precision and personalized medicine such as through the use of multi-omics to resolve heterogeneity in specific cell types contributing to the disease pathogenesis of neuroinflammation and neurodegeneration through different axes of body-brain interactions.

## 2 Crosstalk between inflammation and metabolic dysfunctions

### 2.1 Alterations of inflammatory signaling pathways

Inflammatory cytokine receptor signaling such as tumor necrosis factor (TNF) and TNF receptor 1 (TNFR1) (25, 26) as well as pattern recognition receptor signaling such as lipopolysaccharide (LPS) and toll-like receptor 4 (TLR4) (27) play key roles in mediating inflammation in the body-brain interaction. Stimulation of both signaling pathways lead to the activation of nuclear factor kappa B (NF-κB) transcription factor that could increase the expression of proinflammatory cytokines and chemokines, and also induces activation of immune cells which propagate inflammation between the peripheral and central nervous systems (25, 26). Administration of a soluble TNF inhibitor (XPro1595) to mice fed on high-fat high sugar diet (HFHS) ameliorated liver metabolic disturbances and gut lipocalin-2 levels. In addition, it decreased insulin signaling impairment in the brain, and attenuated glial activation related neuroinflammation and cognitive deficits associated with HFHS diet (28). A TLR4 inhibitor (TAK-242) inhibited inflammation in the adipose tissue but exerted no significant effects on body weight, adiposity, and a range of metabolic measures. In the brain, obese mice treated with TAK-242 exhibited a significant reduction in microglial activation, improved levels of neurogenesis, and inhibition of soluble amyloid beta (Aβ) levels (29). Recently developed receptor-specific inhibitors for TNFR1 (30–33) and TLR4 (34, 35) may also hold promise to mediate obesity induced inflammation. The crosstalk between inflammation and metabolic dysfunction, such as the interaction between TNF-TNFR1 and LPS-TLR4 pathways with insulin signaling impairment (23, 28), mitochondrial dysregulations (36, 37), and autolysosomal defects (22, 38, 39), may provide more insights to the pathogenesis of obesity-induced neuroinflammation and neurodegeneration through body-brain interaction.

Biological molecules such as fibroblast growth factor 21 (FGF21), which is a stress-inducible endocrine hormone predominantly secreted by the liver (40), regulates metabolic homeostasis and possess anti-inflammatory properties (41). FGF21 activates nuclear factor erythroid 2-related factor 2 (Nrf2) pathway and suppresses NF-κB signaling, thereby reducing oxidative stress (42). Treatment of recombinant human FGF21 (rFGF21) in HFD mice reduced body weight, improved glucose metabolism and insulin resistance in the body, and resulted in reduced expression of inflammatory cytokines TNF and IL-1β. It also suppressed microglia activation in the hippocampus, thereby reducing neuroinflammation and cognitive dysfunction and anxiety-like behavior (43). Treatment with FGF21 in HFD rats improved peripheral insulin sensitivity, hippocampal synaptic plasticity, increased dendritic spine density, restoration of brain mitochondrial function, and reduced apoptosis in brain cells, collectively mitigating cognitive decline (44). nOrexin-A (OX-A) or hypocretin-1, a neuropeptide expressed mainly in the brain hypothalamus as well as in several peripheral tissues such as in the intestinal villi has been shown to regulate inflammatory responses (45). Loss of orexin leads to increased microglial activation, increased pro-inflammatory TNF and the mitochondria-associated enzyme immune responsive gene-1, and cognitive decline, which is worsened upon feeding with HFD (46). In microglial under palmitic acid treatment, OX-A addition reduced pro-inflammatory markers TNF, IL-6, and inducible nitric oxide synthase (iNOS), and reduced hypothalamic neuronal cell death (47). The neuroprotective effect of OX-A is mediated through PKC and PI3K signaling pathways, as well as upregulation of somatostatin receptors, vasoactive intestinal peptide, and endothelin-1 (48). OX-A has also been shown to attenuate peripheral inflammation in the gut and reduce the transport of inflammatory mediators such as LPS from the body to the brain, hence decreasing the propagation of neuroinflammation (49). In sum, TNF inhibitors, rFGF21 and OX-A biological molecules play important roles in attenuating both peripheral inflammation and neuroinflammation.

### 2.2 Restoration of metabolic functions

Obesity-induced neuroinflammation contributes to insulin resistance, and promotion of insulin signaling attenuates neuroinflammation and neurodegeneration (50). Restoration of insulin signaling pathway such as through the usage of anti-diabetic drugs is a strategy to alleviate obesity-induced neurodegeneration (51). Glucagon-like peptide 1 (GLP-1) targeting drugs are insulin sensitizers that have shown promise in clinical trials with AD and PD patients (52, 53). GLP-1 exhibits pleiotropic effects owing to the widespread expression of GLP-1 receptors (GLP-1R) across multiple organs, brain regions such as the hypothalamus and amygdala, and various cell types, including microglia and astrocytes. These receptors play key roles in regulating appetite, reward processing, emotional responses, and neuroprotection (52, 53). GLP-1 modulation increases IR and IRS phosphorylation thus improving brain insulin signaling and reducing long term depression. In addition, GLP-1 reduces microglial activation, reactive astrogliosis, thereby attenuating neuroinflammation and neurodegeneration (52, 53). GLP-1R agonists, approved for the treatment of type 2 diabetes and obesity, have demonstrated neuroprotective effects in neurodegenerative diseases through suppressing neuroinflammation (52). Vildagliptin is an inhibitor of dipeptidyl peptidase-4 (DPP-4), an enzyme that degrades glucagon-like-peptide 1 (GLP-1), and has been used to rescue insulin resistance in pancreatic β-cells with lipid overload (54). Treatment with vildagliptin on HFD rat significantly elevates neuronal GLP-1 level, increased brain insulin signaling and attenuated cognitive impairment (55). In another study comparing combined administration of vildagliptin and energy restriction to vildagliptin only, it was shown that HFD rats with combined administration showed decreased body weight, visceral fat, plasma insulin, and reduced total plasma cholesterol levels. Moreover, the brain insulin sensitivity, mitochondrial function, hippocampal synaptic plasticity and cognitive function were restored (56). Dapagliflozin is sodium-glucose co-transporter 2 inhibitor that has also shown effects in restoring insulin sensitivity and neuroprotection in obesity-induced insulin resistance. In a study comparing the effects of Vildaglipti and Dapagliflozin, it was shown that single treatment of either drug resulted in improved brain mitochondrial function, insulin signaling, apoptosis and prevented cognitive decline. Dapagliflozin showed greater efficacy in enhancing peripheral insulin sensitivity, reducing weight gain, and improving hippocampal synaptic plasticity compared to vildagliptin. Notably, the combination of these drugs exhibited superior efficacy in improving brain insulin sensitivity and reducing oxidative stress than either drug alone. These findings suggest that a combination of both drugs could represent a promising therapeutic approach for neuroprotection in obese insulin-resistant conditions, although further studies are needed to fully understand their synergistic mechanisms (57). Metformin, a widely used drug for insulin resistance, has been shown to restore insulin signaling in the liver of obese mice. Additionally, it enhanced insulin signaling in the hippocampus of HFD-fed mice and improved their cognitive function (58). Obesity-induced inflammation and insulin resistance downregulate mitochondrial fusion and upregulate fission, thereby impairing mitochondrial function and exacerbating neurodegeneration. Hence, targeting mitochondrial dysfunction is a viable strategy. In HFD mice, a combined approach consisting of both mitochondrial division inhibitor 1 (Mdivi-1) and mitochondrial fusion promoter (M1) exerted neuroprotection through balancing mitochondrial dynamics, reducing ROS production and depolarization of mitochondrial membranes, thereby improving mitochondrial function (59). The administration of Mdivi-1 and M1 also reduced Aβ aggregation, tau hyperphosphorylation and ameliorated BBB breakdown due to obesity (59). Other studies also demonstrated the beneficial effects of Mdivi-1 in reducing expression of mitochondrial fission protein Drp-1 in palmitate treated hippocampal neural stem cells of rats (60) and HFD rats (61). Garlic extract (Allium sativum) decreased body weight, visceral fat, plasma cholesterol, and MDA levels in HFD rats, and improved brain mitochondrial function, insulin signaling and cognitive functions in HFD rats (62). More evidence is required to strengthen the notion of crosstalk between inflammation and metabolic dysfunctions (63, 64) and how this interplay leads to the pathogenesis of neuroinflammation and neurodegeneration through body-brain interaction under obesity conditions. While the interplay between inflammation and insulin signaling impairment (65, 66) or mitochondrial dysfunction (67, 68) have been established, the link between inflammation and autophagy or lysosomal defects are emerging pathogenic mechanisms that warrant more investigations (22). Several HFD mouse models have illustrated impairments in autolysosomal functions including lysosomal acidification defects (69, 70), together with indications of inflammation. There are further evidence connecting autolysosomal dysfunction and neuroinflammation (22, 71, 72) although this has not yet been established in the HFD rodent models and is an area for future investigations.

## 3 Modulation of gut-brain/gut-liver-brain axis

### 3.1 Alteration of gut microbiota

Gut microbiota is closely linked to brain function via the gut-brain/gut-liver-brain axis, and alterations in microbiota during obesity can lead to neuronal impairment (13). In addition, disruption of the gut barrier leads to more bacteria or their metabolites entering the liver, which contributes to hepatic disorders and influence neural signaling through the gut-liver-brain axis (13). Hence, balancing the microbiota plays an important role in attenuating obesity-induced neuroinflammation and neurodegeneration. Biotics, including probiotics, prebiotics, synbiotics, and paraprobiotics, have been tested in both animal and human trials. These studies have shown a link between gut microbes and immune biomarkers, resulting in improved overall health (73). Treatment with gut commensal *Akkermansia muciniphila* reduced TNF, IL-1β and IL-6 expression in the hippocampus, and reduced hippocampal microgliosis in HFD mice, thereby restoring neuronal development and synapse plasticity (74). *Akkermansia muciniphila* subtype (*A. muciniphila^sub^*) produced short chain fatty acids and improved the spatial memory and blood glucose control in HFD mice (75). Oral supplementation with probiotic *Clostridium butyricum* greatly reduced inflammatory cytokines within the hippocampus of HFD and fiber deficient mice, improved neuronal and synaptic functions and reduced plasma LPS levels, which can be a key mediator in propagating inflammation along the gut-brain axis (76). Probiotic *Lacticaseibacillus rhamnosus* LB1.5 supplementation on HFD mice lowered inflammatory cytokines IL-6 and glial activation in the cerebral cortex (77). Other supplementations of *Lactobacillus paracasei* (78) and *Bifidobacterium infantis* (79) into obese mice also demonstrated effects in reducing neuroinflammation (i.e., IL-6, TNF, and CD11b levels) (79) and increasing neuronal and synaptic functions through increasing the levels of synaptosomal associated protein 25 (78), brain-derived neurotrophic factor (BDNF) and postsynaptic density protein 95 (PSD95) (78, 79). *Lactobacillus paracasei* also reduced inhibitory phosphorylation of IRS to reduce insulin resistance within obese mouse brains (78).

Probiotics including *Lactobacilli*, *Streptococci*, and *Bifidobacteria*, has been shown to reduce the development of liver and brain-related diseases through the gut-liver-brain axis (80, 81). In a study exploring the supplementation of prebiotic (*Xyloolidosaccharide*), probiotic (*Lactobacillus paracasei HII01*), or synbiotics in HFD mice, all of the three supplementations reduced microglial activation, reduced ROS production, and attenuated brain mitochondrial dysfunction and restored cognitive function (82). Synbiotics have demonstrated efficacy in modulating the immune system and addressing neurological disorders linked to impaired liver function through the gut-liver-brain axis (83). In individuals with obesity, supplementation with probiotics containing *Lactobacillus acidophilus*, *Bifidobacterium lactis*, *Bifidobacterium longum*, and *Bifidobacterium bifidum* has been shown to increase the abundance of beneficial gut bacteria, such as *Bifidobacterium* and *Lactobacillus*, and reduce systemic inflammation (84).

Postbiotics are metabolites and bioactive compounds produced by beneficial bacteria in the gut that can potentially influence brain health by mitigating neuroinflammation and slowing neurodegeneration through the gut-brain axis (85). The secondary metabolites of probiotic bacteria are short-chain fatty acids, vitamins, proteins and enzymes, organic acids like propionic acid and 3-phenyl lactic acid, and intracellular polysaccharides. Gut-derived postbiotics have been shown to restore BBB permeability (86, 87). Propionate was found to rescue LPS-induced impairment in the permeability of brain endothelial monolayers by reducing oxidative stress (88). In other studies, butyrate and propionate improved BBB integrity by regulating the organization of the actin cytoskeleton and increasing the interaction between actin and tight junction protein ZO-1, and also restored inflammation induced mitochondrial dysfunction (89). In addition, gut microbiota-derived metabolites, including SCFAs, secondary BAs, indoles, and PUFAs increase the secretion of neuroprotective GLP-1 (90, 91).

The microbiota diversity within the gut can be affected by the type of food that was consumed. Western diet enriched in high amounts of fat and sugars adversely alters gut microbiota contributing to obesity-induced neurodegenerative states (92), whereas consumption of diets rich in fruits and vegetables can reverse altered microbiota states (93). For instance, consumption of kimchi (94) and supplementation of β-glucan (95) reduced neuroinflammation and decreased gut permeability in obese mice. Kimchi further demonstrated effects in reducing BBB permeability (94). Specifically, β-glucan decreases the number of bacteria related to neurodegeneration in obese mice, increased PSD95 and synaptophysin levels, thereby improving synaptic function and memory (95). β-glucan mimics have also been shown to have anti-inflammatory properties (96). The supplementation of anthocyanins increased species of gut bacteria that produces tryptophan, a precursor for kynurenic acid that can exert anti-inflammatory properties (97). Furthermore, microbiota accessible carbohydrate (98), leucine-restricted diet (99) and intermittent fasting (100) also exhibited anti-neuroinflammatory effects, improved synaptic and neuronal function (98–100), reduction in LPS levels (99), and improvement in gut permeability (98–100). In particular, leucine-restriction reshaped the structure of gut microbiota, through downregulating the *Firmicutes/Bacteroidetes* ratio, reducing the relative abundance of inflammation-related bacteria and increasing short-chain fatty acid producing bacterial genera including *Alistipes*, *Allobaculum*, *Odoribacter*, and *Olsenella* (99).

### 3.2 Bariatric surgery and fecal microbiota transplantation

Surgical and other medical interventions could also reverse gut dysbiosis induced by obesity which can eventually benefit the brain. Roux-en-Y gastric bypass (RYGB) done on HFD rats reduced hypothalamic inflammation as seen by lowered cytokine levels and glial cells activation (101). Furthermore, BV2 microglial cells treated with plasma of HFD rats that underwent RYGB had lower inflammatory states compared to those that was treated without surgery (101). The lowered LPS levels and improvement in gut permeability after RYGB further proves the beneficial effect of bariatric surgery in attenuating obesity-induced neuronal damage via the gut-brain axis (101). Another study shows that two different types of bariatric surgery, RYGB and biliary diversion to the ileum, illustrate positive correlation in reducing neuroinflammation compared obese mice (102). Furthermore, gene encoding for protein-tyrosine phosphatase 1B, which is a negative regulator of insulin signaling, was lowered after RYGB surgery (103). There is also improved glucose uptake and increased level of GLP-1, which is a positive regulator of insulin signaling, after duodenum-jejunum bypass, showing the beneficial effect on insulin signaling within the brain together with improved memory (104). A meta-analysis of existing datasets has shown improved cognition in obese patients with gastric by-pass (105), demonstrating the efficacy of this approach in human subjects.

Transplanting feces containing beneficial microbiota, or fecal microbiota transfer (FMT), from healthy donors to the intestinal tract of recipients (106) is another approach to restore gut dysbiosis (107). HFD mice that obtained fecal transplantation from mice that underwent exercise showed improved cognitive behavior, increased BDNF and tropomyosin receptor kinase B (TrkB) levels and reduced astrogliosis (108). Furthermore, the number of beneficial bacteria, *L. acidophilus*, *L. gasseri*, *Christensenellaceae*, *Bactroidetes*, *and Oscillibacter* was increased in mice with exercise, which may also contribute to the improvement along the gut-brain axis (108). In a study comparing the effect of FMT from RYGB donors and oral butyrate supplementation on 24 male and female subjects with metabolic syndrome, it was shown that the FMT from RYGB donors increased brain dopamine transporter and serotonin transporter binding, due to increased levels of *Bacteroides uniformis* compared to oral butyrate (109). However, there was no effect on body weight and insulin sensitivity. Under obesity, there is increased gut permeability and gut dysbiosis, leading to the circulation of inflammatory mediators and metabolites throughout the body, which promotes systemic inflammation and stress. Hence, greater emphasis on developing therapeutics that could ameliorate obesity-induced gut dysbiosis are needed to reduce obesity-induced inflammation.

## 4 Lifestyle changes

### 4.1 Calorie restriction

Calorie intake, meal frequency, diet quality, and gut microbiome interactions influence metabolic and molecular pathways, regulating homeostasis and inflammation in normal brain aging and CNS diseases (110). In a Brazilian longitudinal study of adult health involving 11,737 participants, it was shown that higher adherence to the planetary health diet was associated with slower memory decline (93). Calorie restriction (CR) of a reduction of 40% calories from control effectively decreased phagocytic markers such as galectin-3, dectin-1, CD16 in the white matter indicative of reduced microglial activation, leading to reduced aging-associated decline in HFD mice (111). CR also attenuated the neuroinflammatory response mediated through the triggering receptor expressed on myeloid cells 2-phosphoinositide 3-kinases-protein kinase B (TREM2-PI3K/Akt) signaling pathway, thereby reducing inflammatory cytokines iNOS, cyclooxygenase-2 (COX-2) and IL-1β in the prefrontal cortex and hippocampus of HFD mice (112). Additionally, CR increased proteins crucial for synaptic function in the brain such as BDNF, PSD95, and synaptophysin (112). CR in the form of intermittent fasting reduced TNF expression, decreased oxidative stress markers, and increased autophagy in the cerebellum of HFD mice (113). In mice lacking Sirt3, a mitochondrial deacetylase, the protective effects of CR on oxidative stress and damage are diminished, suggesting that the reduction in oxidative stress during CR requires Sirt3 (114). CR has been shown to increase Sirt3 activation which lead to mitochondrial protein deacetylation, as well as attenuate microglial activation and neuroinflammation (115–117). Additionally, intermittent fasting reduced HFD-induced astrocytic apoptosis and microglial activation as well as reduced memory deficits (118). Intermittent fasting has been used in elderly with obesity and shown effects in improving cognitive impairments, indicated by improved mini-mental state examination and Montreal cognitive assessment scores (119).

### 4.2 Exercise and other environmental stimuli

Exercise has various beneficial effects in alleviating obesity induced neurodegeneration, including increasing neurotrophic factors such as BDNF and TrkB (120–122), and improving synaptic function (123). When HFD mice are subjected to exercise (i.e., voluntary wheel running), there were reduced IL-1β in the hippocampus (124). In another study using voluntary wheel running as exercise, reduced microglial activation and cerebrovascular and white matter damage was seen in HFD mice (125). Additionally, reduction in inflammatory cytokines TNF, IL-1β, and IL-6, improved mitochondrial function and insulin signaling were seen in the hypothalamus, hippocampus and cortex of HFD mice which underwent voluntary wheel running exercise compared to sedentary mice (126). HFD rats on treadmill exercise improved aberrant brain insulin signaling, reduced hyperphosphorylation of tau protein levels (127) and beta-secretase 1 activity (128), reduced oxidative stress and inflammation, and improved mitochondrial function (129). In a study comparing between 12 weeks of aerobic exercise and 12 weeks of resistant exercise on HFD rats, both exercise regimen reduced neuroinflammation, oxidative stress and improved cognitive function, with no significant difference between them (121). Interestingly, when comparing between the effects of caloric restriction and exercise on HFD rats, it was shown that long-term caloric restriction (16-weeks) has the greater systemic metabolic benefit, including improved synaptic function, neuronal insulin signaling, neurogenesis and increased mitochondrial and autophagic function (123). In addition to lifestyle changes, there are a few therapeutic strategies that leverages on environmental stimuli, such as photobiomodulation, to modulate obesity induced neuroinflammation and neurodegeneration (130, 131). For instance, application of near infrared light therapy over HFD mice head reduced inflammatory cytokines TNF and IL-1β levels, as well as reduced microglia and astrocytes activation (130). Far infrared light has also shown effect in reducing IL-6, IL-1β, and TNF and lower the TLR4 and NF-ĸβ pathway, as well as microglial and astrocytes activation in HFD mice (131). HFD mice with environmental enrichment (i.e., equipped with nesting material, a rotating wheel, plastic tubes, and toys) improved neuronal survival and memory function in hippocampus (132, 133) and prefrontal cortex (132).

## 5 Dietary supplements

### 5.1 Vitamin and trace elements

Supplementation with vitamins and natural dietary supplements has been shown to exert neuroprotective effects by reducing neuroinflammation, insulin resistance, and oxidative stress associated with obesity. The supplementation of Vitamin D to HFD rats reduced TNF levels in hypothalamus (134) and hippocampus (135), IL-6 in hypothalamus and hippocampus (136), IL-1β in hypothalamus (136) and NF-ĸβ in hippocampus (135, 137) and hypothalamus (136). Vitamin D also reduced insulin resistance in the brains of HFD rats (134). A natural dietary supplement (NDS) containing curcuma longa, silymarin, guggul, chlorogenic acid and inulin was shown to reduce the activation of astrocytes, ROS, lipid peroxidation and NF-ĸβ, IL-6 and IL-1β levels in the cerebral cortex of HFD mice, indicative of antioxidant and anti-inflammatory effects (138). NDS also exhibited lower levels of ROS, peroxidation, increased levels of heme oxygenase-1 (HO-1) and increased IR expression (138). Lycopene is another dietary supplement with BBB penetrating properties that was able to decrease IL-1β, IL-6, and NF-κB levels within the hypothalamus of HFD rats (139). Lycopene also greatly increases activities of glutathione (GSH), SOD and catalase (CAT) and decreases malondialdehyde (MDA), a by-product of peroxidation caused by ROS, and hydrogen peroxide in the cerebrum of HFD rats (139).

Trace elements such as zinc, magnesium, iron plays an important role in regulating neuronal function (140) and obesity can lead to their dyshomeostasis thereby contributing to neurodegenerative diseases (141). It has been shown that zinc dietary supplementation can penetrate through the BBB and reduce microglial activation in the cerebral cortex as well as TLR4 expression in the hippocampus in HFD rats, although no significant effects was demonstrated in astrocytes (142). In another study, zinc supplementation downregulated phosphorylated signal transducer and activator of transcription 3 (STAT3), thereby attenuating the Janus kinase 2/signal transducer and activator of transcription 3 (JAK2/STAT3) pathway and reduced inflammatory state in the hippocampus (143). Interestingly, the same study show that female HFD rats were more responsive to zinc treatment compared to male HFD rats, suggesting the need to understand more on sex differences and susceptibility to different treatments (143). Supplementation of magnesium in the forms of magnesium oxide and magnesium picolinate significantly reduced NF-ĸβ levels within the brains of HFD rats, potentially via the upregulation of Nrf2, which is an antagonist of the NF-ĸβ pathway (144). Moreover, magnesium supplementation significantly decreased brain MDA levels and increased antioxidant enzymes SOD, CAT, and GSH levels in HFD rats (144). Magnesium supplementation also increased the PI3K/Akt pathway which restored cognitive impairments in HFD rats (144).

### 5.2 Polyunsaturated fats

Supplementation of eicosapentaenoic acid (EPA) and docosahexaenoic acid (DHA) as forms of omega-3 (ω3) reduced IL-1β and TNF in the hippocampus, striatum and prefrontal cortex in HFD mice (145). ω3 also reduced MDA levels in hypothalamus and hippocampus, even though reversal of antioxidant enzymes were not significant (145). ω3 also partially reversed the impairment in mitochondrial function (145). Another study also showed the anti-inflammatory effects of omega-3 through the reduction of astrocytes activation and TNF and IL-6 expression in the cerebral cortex in HFD rats (146). Similarly, other unsaturated fatty acids such as ω9, stearic acid, flax seed and olive oil also significantly reduced inflammatory states within hypothalamus of HFD rats and mice (147). α-lipoic acid reduced TNF and IL-6 in HFD ovariectomized rats (148) and abscisic acid reduced TNF and microglial numbers in hypothalamus of HFD rats (149). Palmitoylethanolamide is a naturally occurring fatty acid amide and has shown effects in reducing NF-ĸβ, IL-1β, and TNF in the hypothalamus and hippocampus while reducing activation of microglia and astrocytes in HFD mice (150). Interestingly, in a study comparing the effects between n-6 polyunsaturated fatty acids (PUFAs) and n-3 PUFAs, it was shown that HFD diet enriched with n-6 PUFAs has detrimental effects on cognitive function in obese mice through upregulating toll-like receptor-myeloid differentiation factor-88-nuclear factor kappa-B (TLR-MyD88-NF-κB) inflammatory signaling pathway, while HFD enriched in n-3 PUFAs has reduced inflammatory responses (151). In another study comparing n-6 PUFAs and n-3 PUFAs, it was shown that the n-6/n-3 PUFAs ratio of 1:1 alleviated inflammatory response and insulin resistance in HFD mice (152). Supplementation of butyrate, a fatty acid produced during microbial fermentation, was able to reduce TNF, IL-1β and IL-6 levels, as well as an increase in anti-inflammatory IL-10 levels in the cerebral cortex of HFD mice (153). Butyrate also protects against oxidative as seen by increased GSH content, state 3 mitochondria respiration and decreased MDA levels (153).

## 6 Biologically derived compounds/bioactive compounds

### 6.1 Compounds derived from biological/natural sources

Bioactive compounds found in naturally derived sources have been shown to have a protective effect against neurodegeneration. Anthocyanins, a class of water-soluble flavonoids widely present in fruits and vegetables, were shown to exhibit various neuroprotective mechanisms (154, 155). Administration of a natural anthocyanin pigment derived from purple sweet potato storage roots to HFD mouse was able to significantly decrease the protein expression of inflammatory cytokines, COX-2, iNOS and NF-ĸβ while increasing anti-inflammatory cytokines in their brains. It was also able to inhibit the activation of mitogen-activated protein kinase (MAPK) pathway which is responsible for inflammation (154). These result in improved locomotor activity and exploratory behavior (154). Blackberry anthocyanin extract decreased expression of cytokines such as TNF and IL-6 in the cortex, thymus and hippocampus of HFD rats (155). Raspberries containing anthocyanins significantly reduced cortical IL-6 levels in HFD mice (156). Peeled extract of *Ananas comosus* (PEAC) (157) (pineapple) reduced IL-6 in brains of HFD rats, reduced MDA levels, and lowered anxiety behaviors and acetylcholinesterase (ACHE) activity (157). Similarly, vegetables such as *Momordica Charanti* (bitter melon) decreased expression of inflammatory cytokines and glial activation within HFD mice brains, reduced oxidative stress, and ameliorated HFD-associated changes in BBB permeability (158). *Cynara Cardunculus* (Artichoke) leaves and *Hericium Erinaceus Mycelium* (mushroom) significantly decreased expression of TNF and IL-1β within the striatum and hippocampus of HFD (159) and HFHS fed mice (160) respectively, leading to improved neuronal survival in the dentate gyrus and spatial memory (160). Compounds like quercetin and catechin in *Momordica charantia*, and luteolin in *Cynara cardunculus*, may play a role in their protective mechanisms. However, further investigations are needed to confirm this. The administration of pomegranate seed oil, juice, fruit and leaves also reduced oxidative stress within rats that were fed a high fructose diet and led to lowered ACHE activity (161).

Green tea is known for its anti-inflammatory and antioxidant properties, which have been studied for their potential in treating neurodegenerative diseases (162). Polyphenol compound extracted from green tea such as epigallocatechin gallate (EGCG) lowered TNF levels through inhibition of the MAPK and NF-ĸβ pathways via oral administration in mice on a high fat high fructose diet (163). In addition, it ameliorated insulin resistance and reduced learning and memory loss (163). Another study using an oral administration of ECGC to inhibit JAK2/STAT3 pathway in microglia, reducing microglial activation and the lowering of TNF, IL-6 and IL-1β levels within the hypothalamus of HFD mice (164). Other polyphenols derived from green tea such as epicatechin (EC) and teasaponin have been shown to reduce microglial activation, TNF and IL-6 levels within the hippocampus and hypothalamus in HFD mice (165). Daidzein, a major isoflavone from soybean, have been shown to lower IL-6 levels in a model of obesity induced neurodegeneration consisting of human fetal hypothalamic gonadotropin-releasing hormone neurons treated with palmitic acid (166). Quercetin, another flavonoid, significantly decreased the levels of TNF, IL-1β and monocyte chemoattractant protein-1 (MCP-1) and microglial activation markers in the hypothalamus of HFD mice (167). This was accompanied by the upregulation of HO-1, which protected against oxidative damage and inflammation (167). Sea-buckthorn flavonoid treatment in high-fat high fructose mice activated ERK/CREB/BDNF and IRS-1/AKT pathways and inactivated the NF-κB signaling (168), thereby preventing neuronal loss and memory impairment. Another flavonoid compound isolated from sea-buckthorn, isorhamnetin, inhibited the phosphorylation levels of JNK, p38, and NF-κβ proteins in the mouse brain, thereby attenuating neuroinflammation and mitigated high fat high fructose-induced cognitive impairment (169).

### 6.2 Compounds derived from medicinal plants

**Figure 3:**
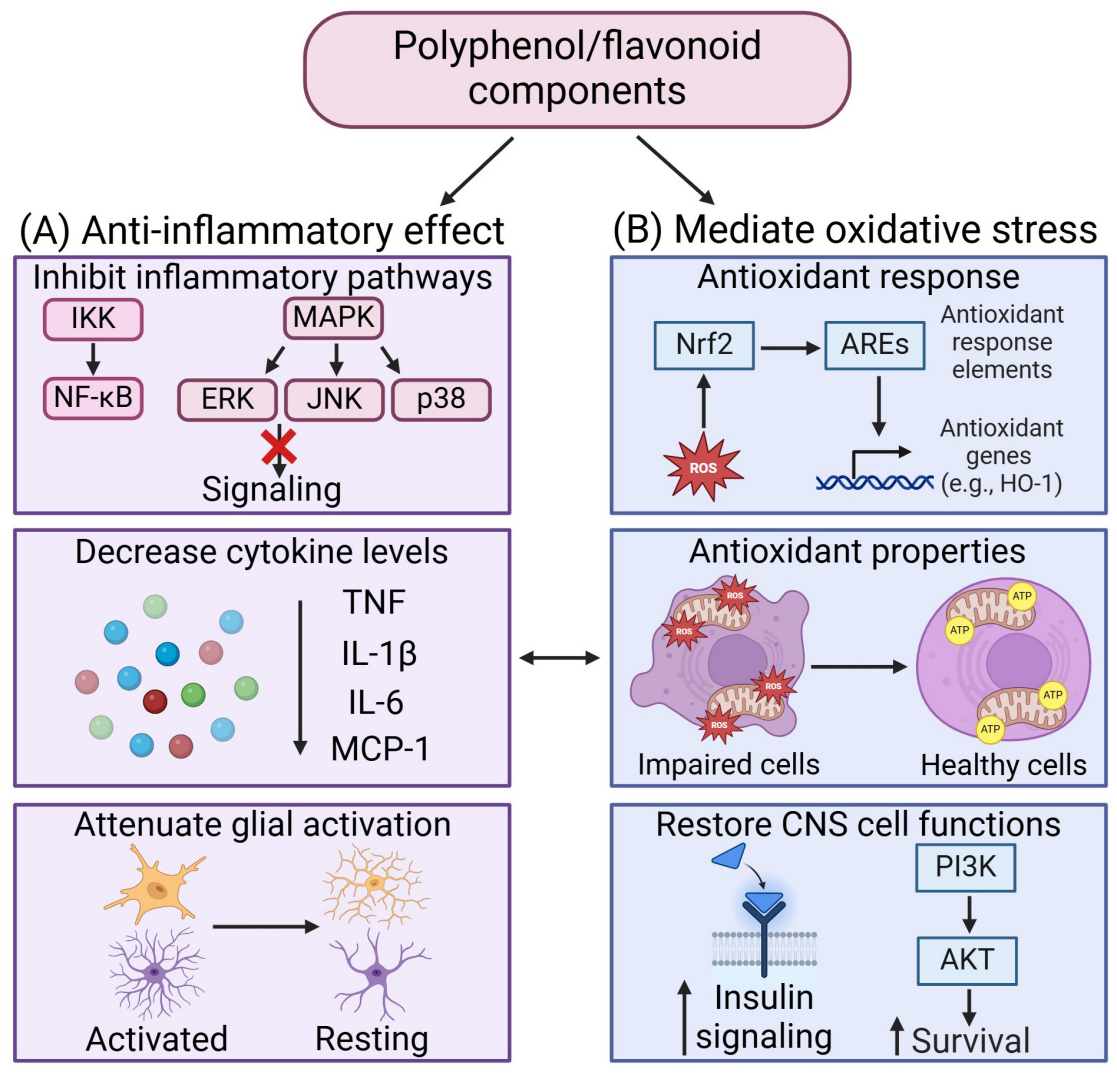
Therapeutic effects of polyphenol/flavonoid in mediating neuroinflammation and oxidative stress. **(A)** The anti-inflammatory effects of polyphenol/flavonoid arise from their ability to inhibit inflammatory signaling pathways, decrease cytokine levels, and attenuate glial activation. **(B)** Polyphenol/flavonoid mediate oxidative stress by activating antioxidant response to enhance antioxidant properties and restore CNS cell functions. Created in https://BioRender.com.

The use of medicinal plants in traditional medicine has consistently demonstrated beneficial effects over time (170). Administration of dry leaf powder of *Withania Somnifera* in HFD rat brains reduced the astrocytic activation in hippocampus and piriform cortex, and microglial activation in the hypothalamus through the attenuation of inflammatory proteins such as iNOS, MCP-1, COX2, NF-ĸβ and cytokines such as TNF, IL-6 and IL-1β (171). Additionally, *Withania Somnifera* reduced insulin resistance through increasing mRNA expression levels of IRS1 and 2 within the hippocampus and piriform cortex (171). Similarly, *Tinospora cordifolia* (herbaceous vine of the family Menispermaceae, traditional ayurvedic medicine) and *Astragalus membranaceus* (Huang Qi, traditional Chinese medicine) also demonstrated effects in reducing glial cells activation within the hippocampus and prefrontal cortex in HFD rats (172) and HFD fed-streptozotocin rats) (173) respectively. *Aruncus Dioicus* var. *Kamtschaticus* (dwarf goat’s beard) and *Mori Cortex Radicis* (Morus alba root cortex, traditional Chinese medicine) protected HFD mice against oxidative stress by increasing antioxidant enzymes (i.e., GSH, SOD and CAT), reducing ROS and MDA levels within their brains. Both compounds were also able to increase IRS-1/AKT insulin signaling in the brains of HFD mice (174, 175). *Curcuma Amada* (Mango ginger) administration attenuates the reduction of antioxidant enzymes levels together with decreasing MDA in the hippocampus of HFHS rats (176). Additionally, *Mucuna pruriens* (velvet bean) have been shown to reduce IL-6 levels in HFD rats and a reduction in depressive behavior (177). *Vigna angularis* (adzuki bean) also reduce inflammation and improve cognitive function in HFD mice (178). Thymol, a monoterpene phenol isolated from medicinal herbs, has exhibited neuroprotective effects through decreasing inflammatory cytokines level TNF and IL-1β, decreasing oxidative stress, and increasing the expression of Nrf2/HO-1 pathway (179). The therapeutic effects of these compounds in mediating neuroinflammation and oxidative stress have been summarized in **Figure 3**.

## 7 Summary and future perspectives

Recent advancements in nanotherapeutics and nanomedicine have increasingly been applied to address obesity-induced neuroinflammation and neurodegeneration. For example, garlic exosome-like nanoparticles have demonstrated the ability to inhibit both systemic and brain inflammation, enhance memory function, and improve glucose and insulin responses in mice with high-fat diet (HFD)-induced obesity through oral administration (180). Similarly, gold nanoparticles administered to HFD mice have been shown to reduce inflammatory markers and oxidative stress in the brain (181). Other types of nanoparticles either target gut dysbiosis (180, 182, 183) or promote autolysosomal functions in the liver and pancreas (69, 70, 184) and potentially exhibit beneficial effects to the brain via the different axes of body-brain interactions. Some of these nanoparticles are also able to partially cross the BBB to target the brain (70, 180, 183, 185) and can be further designed to include theranostic functions (186, 187) as well as improve efficacy in attenuating neuroinflammation and neurodegeneration (185). It is important to note that therapies that do not cross the BBB can still be valuable for testing the effects of preventing neuroinflammation and neurodegeneration in obesity by targeting the peripheral system alone (23). However, therapies that circulate through both the peripheral and central systems are likely more effective, as they can target both body-to-brain and brain-to-body pathways, offering broader therapeutic benefits. Vagus nerve stimulation can also exert neuroprotective effects (188) as well as control satiety which can be a potential therapy to modulate obesity (189).

Given the involvement of multiple organs and cell types in mediating interactions, it is crucial to dissect gene or protein level changes in specific cell types to target the pathogenesis of neuroinflammation and neurodegeneration under obesity conditions. Inflammatory cells such as macrophages and brain glial cells are key targets for modulating inflammation and its spread across organs and cell types, which can lead to neuronal death. To advance precision and personalized medicine, utilizing multi-omics technologies like metabolomics and microbiome sequencing (190, 191), as well as data mining of existing datasets (192, 193), is essential for elucidating disease mechanisms and developing more effective targeting strategies.
